# Endovascular vs conservative treatment in patients with chronic subdural hematomas and mild symptoms: a study protocol for a multicenter randomized controlled trial (EMBOTRIAL-1)

**DOI:** 10.1186/s13063-025-09131-y

**Published:** 2025-11-18

**Authors:** Giancarlo Salsano, Pietro Fiaschi, Giorello Laura, Mammoliti Sonia, Valeria Fontana, Luca Boni, Gianluigi Zona, Lucio Castellan

**Affiliations:** 1 Department of Radiology and Interventional Radiology, Neuroradiology Unit, IRCCS Ospedale Policlinico San Martino, Largo Rosanna Benzi, 10, 16132 Genoa, Italy; 2 Neurosurgery Unit, IRCCS Ospedale Policlinico San Martino, Genova, 16132 Italy; 3 Clinical Epidemiology Unit, IRCCS Ospedale Policlinico San Martino, Genova, 16132 Italy; 4 Management of research activities and Grant Office, Galliera Hospital, Genova, 16128 Italy

**Keywords:** Chronic subdural hematoma, Middle meningeal artery, Embolization, Best medical treatment, Mild symptoms

## Abstract

**Background:**

Chronic subdural hematoma (cSDH) is a common neurosurgical condition that is prevalent in elderly patients. There are no well-defined guidelines for cSDH and management strategies vary widely among physicians and institutions, and this variability is expressed in the literature. In general, conservative management is reserved to patients who are asymptomatic or have minor symptoms with mild mass effect. Spontaneous resolution of cSDH is an unusual phenomenon and middle meningeal artery (MMA) embolization seems to reduce the recurrence and progression rate of SDH compared to conventional treatments in multiple cohort studies. A randomized controlled trial is warranted to determine the effectiveness and safety of endovascular embolization for cSDH and whether MMA embolization is superior to conservative management in reducing the progression rate and surgical rescue event.

**Methods:**

This is an Italian multicenter prospective randomized clinical trial with open-label treatment and blinded outcome assessment (PROBE design) to assess the superiority of MMA embolization compared to conservative treatment. A total of 300 patients are planned to be randomized 1:1 to receive MMA embolization (intervention) or conventional treatments (control). The primary outcome is the treatment arm failure which is defined as a composite of incomplete hematoma resolution or surgical rescue within 6 months follow-up. Incomplete hematoma resolution is defined as a reduction of the cSDH thickness ≤50% at follow-up compared to the hematoma thickness measured at the time of randomization. Surgical rescue is intended as hematoma removal for relief or symptoms that developed with continuous growth of the cSDH. In case of bilateral cSDH, the treatment failure occurred when primary outcomes criteria are satisfied for at least one of the two hematomas.

**Discussion:**

This multi-centre randomized controlled trial is needed to evaluate the benefit-to-risk ratio of primary embolization of the MMA to facilitate resolution and prevent rescue surgical evacuation of cSDH. If MMA embolization turns out to be superior to conservative management in this trial, this may prompt further confirmatory trials and, at best, may change clinical practice and guideline recommendations.

**Trial registration:**

ClinicalTrials.gov. Identifier: NCT06274580, Registered on 6 February 2024. This protocol was developed in accordance with the SPIRIT Checklist and by use of the structured study protocol template provided by BMC Trials.

**Supplementary Information:**

The online version contains supplementary material available at 10.1186/s13063-025-09131-y.

## Administrative information

**Table:** 

Title {1}	Endovascular vs Conservative Treatment in Patients with Chronic Subdural Hematomas and Mild Symptoms: a Study Protocol for a Multicenter Randomized Controlled Trial (EMBOTRIAL-1).
Trial registration {2a and 2b}.	ClinicalTrials.gov Identifier: NCT06274580.
Protocol version {3}	Date: 16/01/2023, Version 1.0.
Funding {4}	No profit trial.
Author details {5a}	Giancarlo Salsano, Pietro Fiaschi, Giorello Laura, Mammoliti Sonia, Valeria Fontana, Luca Boni, Gianluigi Zona, Lucio Castellan.
Name and contact information for the trial sponsor {5b}	Giancarlo Salsano^1*^, Pietro Fiaschi^2^, Giorello Laura^3^, Mammoliti Sonia^4^, Valeria Fontana^3^, Luca Boni^3^, Gianluigi Zona^2^. Lucio Castellan^1^; on behalf of EMBOTRIAL-1 investigators. Affiliations: ^1^ IRCCS Ospedale Policlinico San Martino, Neuroradiology Unit, 16132, Genova. ^2^ IRCCS Ospedale Policlinico San Martino, Neurosurgery Unit, 16132, Genova. ^3^ IRCCS Ospedale Policlinico San Martino, Clinical Epidemiology Unit, 16132, Genova. ^4^ Galliera Hospital, Management of research activities and Grant Office, 16128 Genova *Correspondence: giancarlo.salsano@yahoo.it
Role of sponsor {5c}	Not applicable.

## Introduction

### Background and rationale {6a}

Chronic subdural hematoma (cSDH) is among the most common reasons for cranial neurosurgical consult with a reported annual incidence ranges from 14.1 to 20.6 per 100 000 person [1]. It becomes more frequent in elderly patients with an occurrence of 58.1/100000 [2]. Moreover, the wide use of computed tomography (CT) and magnetic resonance imaging (MRI) contributed to increase cSDH detection in the last years.

The pathogenesis of cSDH comprises the formation of fragile capillaries along the subdural neo-membrane encapsulating the blood collection [3]. Risk factors associated with cSDH development are older age, male gender, craniocerebral disproportion, anticoagulation and antiplatelet therapy, bleeding disorder, alcohol abuse, diabetes mellitus and arterial hypertension [4–6]. The cSDH cannot be considered a benign condition, as approximately 30% of patients have a poor prognosis requiring some help at discharge with greater likelihood in aging people [2].

Non-operative management with best medical therapy and serial cranial CT follow-up is the standard treatment for patients affected by cSDH with the following characteristics: a) few symptoms and Markwalder’s Grading Scale-Glasgow Coma Scale (MGS–GCS grade) 0–2 [7]; (b) image showing low mass effect with midline shift less than 1 cm (c) patients having contraindications or refused surgeries [8].

Surgical evacuation (burr-hole drainage or craniotomy) is reserved to patients with symptomatic cSDH having significant mass effect [9].

Natural history of asymptomatic cSDH is not well established and spontaneous resolution of subdural blood collection is reported in literature between 3% and 18% [10, 11]. Several pharmacological therapies have been studied to improve resolution rate, such as corticosteroid [12], angiotensin converting enzyme (ACE) inhibitors [13] and statins [14]. However these reports, have failed to demonstrate their efficacy on hematoma reabsorption with safety concerns [12–14]. With the increasing complexity of the modern patient with cSDH, the embolization of middle meningeal artery (MMA) has emerged as a promising novel treatment. The endovascular approach by eliminating the vascular supply to the subdural neo-membrane seems to reduce progression and recurrence rates of cSDH [15].

Recent systematic review and meta-analyses [16, 17] reported that MMA embolization was associated with lower rates of cSDH recurrence and surgical rescue and higher hematoma resolution rate comparing with conventional treatment.

The randomized trial of endovascular approach (EMBOTRIAL-1) aims to assess the efficacy and safety of MMA embolization over conservative management as primary treatment in patients with cSDH and mild symptoms.

### Objectives {7}

#### Primary objectives

The primary objective of this study is to investigate whether endovascular embolization of MMA is superior in terms of clinical and radiological outcome compared to conservative management.

#### Secondary objectives

Secondary objectives of this study include assessment of:

- Endovascular procedure related complications;
- Neurological deficits;
- Recurrence rate associated with endovascular embolization of MMA;
- Resolution rate.

### Trial Design {8}

**Fig. 1:**
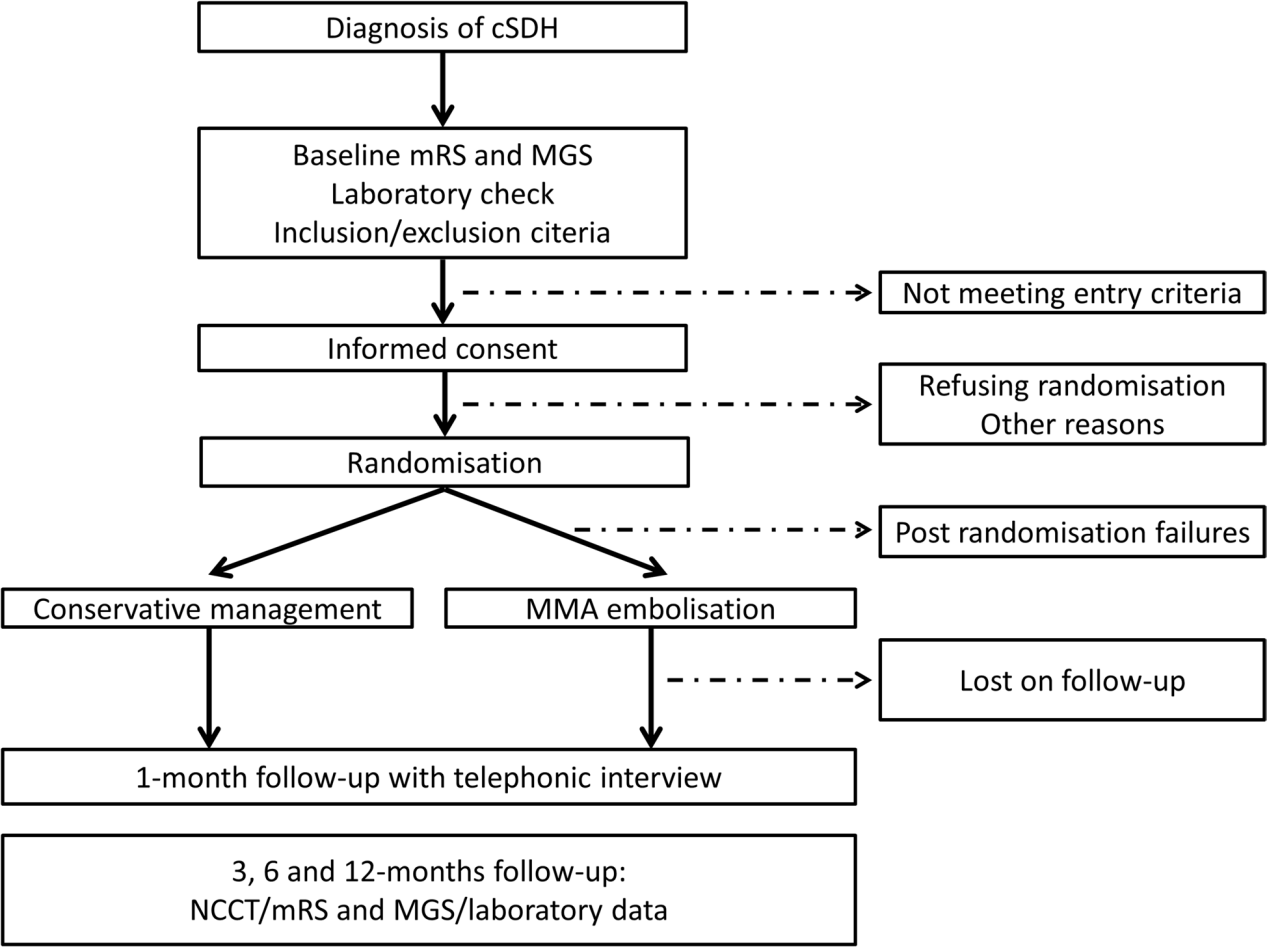
The flow diagram displays the main study processes, assessments and interventions, including follow-up evaluations

Embotrial-1 is an Italian multicenter prospective randomized clinical trial with open-label treatment and blinded outcome assessment (PROBE) to assess the superiority of MMA embolization compared to conservative treatment. The intervention group is MMA embolization and comparator control group is the conservative management and best medical treatment. Patients are randomized 1:1. Patient flowchart is depicted in Fig. 1.

## Methods: participants, interventions and outcomes

### Study setting {9}

The study is not funded. As a quality standard, all the Italian centers involved should meet the following minimum criteria: (1) local tertiary hospital, (2) experience in managing brain trauma or neurovascular disease trials, and (3) could carry out MMA embolization and have more than 30 cases of cSDH every year. Currently, 7 centers (Supplementary material 1) in Italy have been identified for the study. All the clinical investigators are board-certified and have renowned expertise in neurosurgery, neuroradiology and endovascular interventions. The study is open to additional participating neurosurgical centers.

### Eligibility criteria {10}

#### Inclusion criteria

Patients can be included in Embotrial-1 if they meet these criteria:

- Age over 18 years;
- No neurological deficits (Markwalder score ≤1);
- Unilateral or bilateral cSDH;
- Subdural Hematoma width ≤ 20 mm; midline shift ≤ 7 mm;
- Independent functional status with mRS score ≤ 2 prior to symptom onset;
- Written informed consent of the patient to participate in the trial;
- Likely compliance of the participant in attending follow-up examination.

#### Exclusion criteria

The exclusion criteria are the following:

- Radiological evidence of an acute or subacute subdural hematoma, subarachnoid hemorrhage, intracerebral hematoma or epidural hematoma;
- Contraindications to angiography (end stage chronic renal disease, any sign of anatomical variations that could make MMA embolization unsafe, pregnancy);
- Life expectancy < 1 year;
- Patients with any kind of ventricular derivation catheter.

### Who will take informed consent? {26a}

Investigators or their representatives being part of Department of Neurosurgery or Department of Radiology and Neuroradiology will have to adequately explain the details of the clinical trial including rationale, design, risks and potential benefits of the study. Patients will be given ample time to think about the opportunity to participate and to discuss the study with their family and their general practitioner. After full and detailed explanation, subjects or their guardians, as well as the investigators, will sign their name and date on the informed consent form.

### Additional consent provisions for collection and use of participant data and biological specimens {26b}

N/A. Biological specimen collections are not needed in this trial.

### Interventions

#### Explanation for the choice of comparators {6b}

Patients assigned to the control group will managed according to the current standard of care with “wait and see” approach and best medical treatment.

#### Intervention description {11a}

Patients randomized to the experimental arm will submit to cerebral angiography and bilateral MMA embolization of the cSHD, even if it is located to only one side. The ipsilateral MMA embolization of the cSDH is allowed when the endovascular procedure cannot be performed on both sides. Patients are preferably under monitored local anesthesia or alternatively under conscious sedation or general anesthesia. Briefly, through femoral or radial artery access a standard 5 French diagnostic catheter is placed in the proximal external carotid artery and a digital subtraction angiography (DSA) is performed. Then, a microcatheter is advanced selectively under roadmap guidance into the main trunk of MMA, and superselective angiography is performed to evaluate for potentially dangerous anastomoses between MMA and ophthalmic or petrous branches prior to embolization [18]. After collateral vessels are excluded, the MMA is occluded with PVA particles (sizes between 40-300µm) or liquid embolizing materials (Onix^®^, Squid^®^, Phil^®^, glue). The successful embolization is defined as that both frontal and parietal branch of MMA are selectively occluded and for this purpose, the microcatheter should be placed as distally as possible. It is recommended to ensure embolic materials penetrating into the capillary network of the dura to be permanently blocked [19]. The use of coils should be avoided because a faster reperfusion via collaterals might be possible.

#### Criteria for discontinuing or modifying allocated interventions {11b}

Patients may withdraw their consent at any time without providing a reason and thus terminate their participation in the study prematurely. Withdrawal from the study and reasons, if known, will be documented. Criteria for premature drop-out include the following: subsequent occurrence of an exclusion criterion, loss of contact, death, and declaration of withdrawn consent.

#### Strategies to improve adherence to interventions {11c}

To enhance participant adherence to the study, the following strategies were implemented.

- Participant training: Clear and accessible instructions will be provided both verbally and in written form at enrollment and prior to each intervention session.
- Reminders: Email and/or telephone reminders will be sent 24 hours before each intervention session.
- Support mechanisms: Participants will have access to a study coordinator who will be available to address any questions or concerns related to the intervention.
- Motivational engagement: Periodic check-ins will incorporate motivational interviewing techniques to support sustained engagement throughout the study.

#### Relevant concomitant care permitted or prohibited during the trial {11d}

Patients in both groups are treated according to the currently established standard of care at the trial center. Any concomitant care as part of routine clinical practice is permitted.

#### Provisions for post-trial care {30}

A proband cover for all patients participating in the study is contracted to compensate for trial-associated harm occurring within 3 years of trial participants’ final study visit.

### Outcomes {12}

#### Primary Outcomes

The primary outcome is the treatment arm failure which is defined as a composite of incomplete hematoma resolution or surgical rescue within 6 months follow-up. Time zero is defined as the date of randomization for both groups in order to ensure comparability and avoid immortal time bias. For patients randomized to the intervention arm, any surgical rescue occurring after randomization but before the scheduled embolization procedure will be considered a treatment failure, and will be included in the intention-to-treat analysis.

Incomplete hematoma resolution is defined as a reduction of the cSDH thickness ≤50% at follow-up compared to the hematoma thickness measured at the time of randomization.

Surgical rescue is intended as hematoma removal for relief or symptoms that developed with continuous growth of the cSDH.

Symptoms onset were evaluated from clinical investigators at follow-up based on Modified Rankin Scale Score (mRS) [20] and Markwalder scale [7]. In particular, patients with mRS≥3 and Markwalder scale≥2 are candidates to surgical evacuation of cSDH.

#### Secondary outcomes

The following secondary outcomes will be assessed:

- Successful embolization rate of the target vessels based on DSA.
- Complete resolution of cSDH at 6 months follow-up;
- Recurrence rate of cSDH at 12 months follow-up;
- Procedure related complication during or 30 day after MMA embolization;
- Adverse event related to conservative management 30 day after randomization.

#### Participant timeline {13}

All trial procedures including radiological and clinical visits are summarized in Table 1.

**Table 1:** Schedule of enrollment, interventions and assessments

**Timing of all procedures in EMBOTRIAL-1**						
**Timepoint**	-t_1_	t_0_	1 month	3 months	6 months	1 year
**Enrollment**						
Eligibility screen	X					
Informed consent	X					
Randomization		X				
**Interventions**						
MMA embolization		X				
Conservative treatment		X				
**Assessments**						
Demographics data﻿	X					
Medical history	X					
Laboratory data	X			X	X	X
Non contrast CT	X			X	X	X
mRS/Markwalder scale	X			X	X	X
DSA imaging		X				
EVT-Complications		X	X			
Adverse events			X	X	X	X

#### Sample size {14}

Our estimates are based on treatment failure rates of previous published studies. Ban et al. [18] compared a prospective series of 72 patients with cSDH submitted to MMA embolization with a group control of 469 subjects undergoing standard treatment. In particular, in the amount of patients into embolization group, 27 (37.5%) were asymptomatic and underwent MMA embolization alone. No treatment failure was found during follow-up after embolization. Of 469 patients in the control group, 67 patients underwent close follow-up. Spontaneous resolution occurred in 11 (16.4%), but treatment failure with surgical rescue was needed in 56 (83.6%) for symptom development resulting from progression of hematoma. Moreover, Catapano et al. [21] reported a treatment failure rate of 17% and of 3%, respectively with conservative approach and MMA embolization. Other studies reported progression rate of conventional treatment ranged from 11 to 27.5% [22–24]. So we assume the event rate of the intervention group is 1%. The trial is powered to assess superiority. When assuming the event rate of 1% in intervention group and 10% event rate in the control group, with a power of 85% and two-sided alpha of 0.05, allowing for 10% drop-outs, the estimated sample size is 300 patients in total.

#### Recruitment {15}

During the kick-of meeting, the clinical investigators of each center will be trained in communicating with potential participants and their relatives, documentation including screening logs, and other standard operating procedures. All the centers will recruit patients competitively, and recruitment progress will be reported to the steering committee at 6, 9, 15, 21 months after randomization of the first patient, to track the recruitment process. The estimated rate of recruitment is 3 to 5 patients per month in high volume centers and 1–2 patients in medium-low volume centers, and the expected recruitment time will last 2 years. In case of insufficient enrollment (i.e., <25% of the number of patients included at the fixed time points), investigators will take extra measures to increase recruitment (e.g., increasing awareness among trial supporting personnel, include other institutions, etc.).

### Assignment of interventions: allocation

#### Sequence generation {16a}

After inclusion criteria of patients with cSDH has been satisfied and written informed consent form has been obtained, randomization is allowed. Patients will randomly be assigned to trial arms using a web platform and an extended stratified block algorithm. The algorithm guarantees even distribution of key baseline characteristics across the intervention and the control group.

#### Concealment mechanism {16b}

Participants are randomized using the Research Electronic Data Capture (REDCap), a web-based, GCP compliant electronic data capture (EDC) system for collecting patient data in clinical trial, observational studies, and registries. The software REDCap is a secure, browser-based web application available to approximately 2.3 million users in 151 countries [25]. It maintains allocation concealment as it does not release the randomization code until screening has been completed and the patient was cleared to be recruited onto the trial.

#### Implementation {16c}

Extended stratified block algorithms generate an unpredictable allocation sequence. Random assignment by REDCap [25] cannot be influenced by clinical investigators.

### Assignment of interventions: blinding

#### Who will be blinded {17a}

Due to open-label trial design, the treatment allocation is known to both the treating physician and the patient. In each center is nominated a blinded expert physician that will procure the information concerning clinical outcome at 3, 6 and 12 months. Radiological assessment is also performed by blinded and trained neuroradiologist evaluating non contrast CT scan (NCCT) after 3, 6 and 12 months from randomization. To report to the data safety monitoring board (DSMB), outcome data will be combined with data on treatment allocation by an independent trial statistician. Members of the steering committee are kept blinded of results of interim analyses of efficacy and safety.

#### Procedure for unblinding if needed {17b}

N/A. We do not anticipate any requirement for unblinding but if required, the Trial Manager, Data Coordinator, Implementation Support Facilitators and Care Home Managers will have access to group allocations and any unblinding will be reported.

### Data collection and management

#### Plans for assessment and collection of outcomes {18a}

Data will be entered by physician and supporting trial personnel on electronic case report forms (eCRFs). Clinical examination comprises the mRS and Markwalder scale for neurologic disability. Standard laboratory parameters will be taken from peripheral blood at baseline and during planned follow-up. The size and extent of cSDH will be measured on the coronal scans of NCCT, at the point of maximum thickness of the blood collection.

#### Plans to promote participant retention and complete follow‑up {18b}

Investigators, doctors, and nurses will take very care of all trial participants until the scheduled last follow-up visits. Patients will also be explained there is scientific evidence that patients included in a clinical trial environment may show superior outcomes compared to those managed under routine practice conditions.

#### Data management {19}

All patient data is registered in the electronic data capture software REDCap. This worldwide online system allows built-in logical checks and validations to promote data quality. All clinical data are entered via an encrypted connection, are anonymized, and fulfill the demands for data protection. All data entries and changes are logged in REDCap [25] and meet the Good Clinical Practice (GCP) requirements for the use of the electronic case report form (eCRF) in medical trials. Trial coordinators, data managers and the investigators will be introduced to the platform and trained in data entry during the initial kick-of meeting prior to recruitment of the first patient. Trial staff will be provided with a personal ID.

#### Confidentiality {27}

All study-related information will be stored securely in every participating center. All participant information will be kept in a locked space to which only the principal investigator has access and may be used to unblind personal data if necessary. Data processing and statistical work will be performed by exporting the data from REDCap to a secure server. Participant confidentiality will always be maintained.

#### Plans for collection, laboratory evaluation and storage of biological specimens for genetic or molecular analysis in this trial/future use {33}

N/A as no biological specimen is collected as part of this trial.

### Statistical methods

#### Statistical methods for primary and secondary outcomes {20a}

Categorical data were presented as frequencies and percentages and compared using the Chi-square test or Fisher’s exact test where appropriate. Continuous variables were expressed as median, deviation standard [DS] and interquartile range [IQR] and compared using a two-tailed Mann–Whitney test for non-parametric distributions. The primary outcome analysis is to evaluate treatment failure rate of mild symptoms cSDH (Markwalder scale 0−1) within 6 months follow-up post randomization and to determine whether MMA embolization is superior to conservative management. Analysis will be performed on an ‘intention-to-treat’ principle. The chi-square test or Fisher’s exact test will be used as primary analysis to test the difference in primary outcome between the experimental and control groups. Moreover, odds ratio (OR) with 95% confidence intervals will be calculated by a logistic regression model. Binary second efficacy outcomes will be analyzed using the same method as primary endpoint analysis. Procedure-related complications and (serious) adverse events (AEs/SAEs) will be expressed as absolute and relative frequencies.

#### Interim analyses {21b}

The safety and efficacy interim analysis will be planned when half of the target sample (150 patients) have completed 6 months follow-up. The enrollment will be stopped for futility if the estimated conditional power is <0.7% (i.e., <0.007 probability). This highly conservative threshold was selected in agreement with the independent biostatistician and the DSMB to limit premature termination of the study, and ensures that the trial will only be stopped for futility in the unlikely event that accumulating data indicate virtually no chance of demonstrating a treatment effect.

#### Methods for additional analyses (e.g., subgroup analyses) {20b}

Primary and secondary outcomes will be stratified in a categorical manner for age (≤60 and >60 years), arterial hypertension, anticoagulant and antiplatelet home therapy and ipsilateral or bilateral side of hematoma.

#### Methods in analysis to handle protocol non-adherence and any statistical methods to handle missing data {20c}

The primary analysis will be performed on the intent-to treat set including all randomized patients and based on the treatment arm they were randomized to, regardless of the therapy they actually received. A missing data analysis will be conducted investigating the extent and type of missing values in trial endpoint variables. In case of a suspected non-random missing data mechanism, corresponding sensitivity analyses will be carried out along with a discussion of the results. If suitable, we will consider multiple imputations to full empty cells in the dataset.

#### Plans to give access to the full protocol, participant level-data and statistical code {31c}

The trial was registered prospectively in ClinicalTrials.gov on 6 February 2024 with the Identifer NCT06274580

### Oversight and monitoring

#### Composition of the coordinating center and trial steering committee {5d}

The Coordinating Centre and trial steering committee will comprise clinical trial managers, data managers, statisticians, clinical researchers, regulatory specialists and administrative staff.

### Roles and responsibilities of coordinating centre and trial steering committee

#### Coordinating Centre

- Manage the day-to-day operations of the trial
- Ensure regulatory and ethical compliance
- Coordinate site initiation, monitoring, and close-out activities
- Manage data collection, cleaning, and validation
- Handle communication with trial sites and oversight committees
- Maintain the trial master file and ensure proper documentation
- Provide logistical and organizational support to all trial stakeholders

#### Principal investigators

- Preparation of protocol and revisions;
- Preparation of written patient information;
- Applying for ethical approval;
- Organizing steering committee meetings;
- Publication of study reports.

#### Steering committee

- Agreement of final protocol;
- All investigators will be steering committee members; one lead investigator per department will be nominated as the local coordinator;
- Recruitment of patients;
- Reviewing the progress of the study and, if necessary, agreeing on changes to the protocol;
- Data verification;
- Randomization.

#### Data manager

- Maintenance of the trial IT system (REDCap);
- Monitoring of all activity in the REDCap system;
- Data verification.

### Person responsible for data monitoring

An independent biostatistician will perform all the statistical analyses throughout the study.

#### Composition of the data monitoring committee, its role and reporting structure {21a}

A Data Safety Monitoring Board (DSMB) will regularly receive blinded statistical reports and monitor serious adverse events (SAEs) throughout the trial, decide whether patient safety is compromised, demanding premature closure of the trial. The DSMB plans to perform the safety analysis when randomization and 6 months follow-up is completed of the first 150 patients. An ad hoc meeting of the DSMB may be called at any time by the principal investigators or the DSMB if imminent participants’ safety issues arise.

#### Adverse event reporting and harms {22}

Adverse events (AEs) and serious adverse events (SAEs) are defined according to the ICH GCP guidelines [26]. The severity of AEs will be assessed according to the Common Terminology Criteria for Adverse Events (CTCAE) [27], which is a 5-point scale designed for use in clinical trial. It goes from Grade 1 (mild, asymptomatic or with few associated symptoms, clinical observation, no intervention required, preventive treatment if available) to Grade 5 (death) [27]. All AEs and SAEs reported by study participants or observed by an investigator within the study period must be documented in the eCRF and be reported to DSMB. The causal relationship between the MMA embolization and AEs will be investigated further, and patients will be observed until the AEs resolve or a stable condition is reached.

#### Frequency and plans for auditing trial conduct {23}

The Project Management Group (PMG) will meet monthly to review trial conduct, monitor recruitment, adherence to the protocol, data quality, and address any operational issues.

The Trial Steering Committee (TSC) will meet approximately every 6 months to provide overall supervision of the trial, ensuring that it is conducted in accordance with the protocol, Good Clinical Practice (GCP), and relevant regulations.

Data management staff will stay in regular contact with steering committee about trial progress, data consistency, missing data and time window violations. If necessary, data queries for missing data as well as clarifications of inconsistencies or discrepancies will be sent.

#### Plans for communicating important protocol amendments to relevant parties (e.g. trial participants, ethical committees) {25}

All proposed amendments to the protocol will first be reviewed and approved by the ethical committee. Once approval has been obtained, the Chief Investigator (CI)/Principal Investigator (PI) will be responsible for notifying all participating study centres of the changes. A copy of the revised protocol will be distributed to each site’s Principal Investigator, who will ensure that it is filed in the Investigator Site File (ISF) and that all relevant site staff is informed of the changes. Any deviations from the approved protocol will be fully documented using a Breach Report Form, in accordance with Good Clinical Practice (GCP) and regulatory requirements. The protocol will also be updated in the relevant clinical trial registry (e.g., ClinicalTrials.gov or ISRCTN) to ensure transparency and regulatory compliance.

#### Dissemination plans {31a}

After database closure, a biometric report will be written by the trial statistician describing the main study results. Subsequently, a meeting among investigators and collaborators will be held to discuss findings prior to drafting of a scientific manuscript to be submitted for peer-review and publication in a major scientific journal. Also, we will attempt to present results at key international conferences of neurosurgical and neurointerventional societies.

## Discussion

Embotrial-1 is a randomized clinical trial with a PROBE design assessing superiority of MMA embolization compared to conservative management. Without clinical trials, the best evidence for primary treatment of asymptomatic cSDH with MMA embolization is limited to observational studies and meta-analyses which have shown promising results on reducing progression and recurrence rate of blood collection without major concerns. This randomized controlled trial is needed to evaluate the beneft-to-risk ratio of primary embolization of the MMA to facilitate resolution and to prevent reaccumulation with consequent reduction of rescue surgical evacuation of cSDH. If MMA embolization demonstrates superiority over conservative management in this trial, it could potentially lead to further confirmatory studies and, depending on the strength of the evidence, might influence clinical practice and guideline recommendations.

A methodological limitation of the study is the potential occurrence of surgical rescue between randomization and MMA embolization in the experimental group. To address this, randomization was chosen as the starting point of follow-up for both study arms, and any surgical rescue before embolization will be considered as treatment failure in the intention-to-treat analysis. This approach minimizes immortal time bias but may lead to an overestimation of treatment failures in the intervention arm.

### Trial status

This manuscript is based on trial protocol version 1.0, dated 16 January 2023. First patient has been randomized on 7 March 2024. Currently (12^th^ of June 2024) we included 8 patients. Recruitment is estimated to be completed 24 months after.

## Supplementary Information

Supplementary Material 1.

## Data Availability

Data will be made available from the corresponding author upon reasonable request.

## Abbreviations

EMBOTRIAL-1: Middle meningeal artery embolization versus best medical treatment for asymptomatic chronic subdural hematomastudy protocol for a randomized controlled trial
cSDH: chronic subdural hematoma
MRI: magnetic resonance imaging
NCCT: non contrast computed tomography
MGS–GCS: Markwalder’s Grading Scale-Glasgow Coma Scale
ACE: angiotensin converting enzyme inhibitors
MMA: middle meningeal artery
mRS: Modified Rankin Scale Score
DSA: digital subtraction angiography
PVA: Polivinil alcohol particles
REDCap: Research Electronic Data Capture
DSMB: data safety monitoring board
eCRFs: electronic case report forms
GCP: Good Clinical Practice
AEs/SAEs: adverse events/serious adverse events
CTCAE: Common Terminology Criteria for Adverse Events
