# Supplementary material for: Endovascular vs conservative treatment in patients with chronic subdural hematomas and mild symptoms: a study protocol for a multicenter randomized controlled trial (EMBOTRIAL-1)
Source: Trials. 2025 Nov 18;26:517. doi: 10.1186/s13063-025-09131-y (PMC12625609; doi:10.1186/s13063-025-09131-y)
Supplement: Supplementary file 1 — Supplementary Material 1. [file 13063_2025_9131_MOESM1_ESM.docx]

**Appendix I.**

**Centres enrolling patients:**

1. Neuroradiology unit and Neurosurgery unit, IRCCS Ospedale Policlinico San Martino, 16132, Genova, Italy;
2. Neuroradiology unit, Azienda ospedaliero-universitaria Bolognese, Bologna, Italy;
3. Neuroradiology unit, Ospedale M. Bufalini, Cesena, Italy;
4. Neuroradiology unit, Ospedale San Gerardo, Monza, Italy;
5. Neuroradiology unit, Azienda ospedaliero-universitaria Senese, Siena, Italy;
6. Neuroradiology unit, Azienda Ospedaliero-Universitaria di Parma, Parma, Italy;
7. Neuroradiology unit, A.O.U. Citta della Salute e della Scienza di Torino, Torino, Italy.
